# Carcinogenic PAHs (BaP and PAH4) in Breast Milk: Dietary and Environmental Determinants Among Hungarian Mothers

**DOI:** 10.3390/toxics14070596

**Published:** 2026-07-07

**Authors:** Timea Dergez, Anditi Bernard Collins, Dénes Szerencsés, István Szabó, Mátyás Wahr, Anikó Kőnig-Péter, Viktória Poór

**Affiliations:** 1 Institute of Bioanalysis, Medical School, University of Pécs, Szigeti út 12, 7624 Pécs, Hungary; timea.dergez@aok.pte.hu (T.D.); collinsanditi2@gmail.com (A.B.C.); szerencsesdeni@gmail.com (D.S.); matyas.wahr@profood.hu (M.W.); viktoria.poor@aok.pte.hu (V.P.); 2 Doctoral School of Chemistry, Faculty of Sciences, University of Pécs, Ifjúság útja 6, 7624 Pécs, Hungary; 3 Department of Environmental Toxicology, Hungarian University of Agricultural and Life Sciences, Páter Károly utca 1, 2100 Gödöllő, Hungary

**Keywords:** benzo[a]pyrene, breast milk, carcinogenic PAHs, environmental exposure, Hungarian mothers, PAH4

## Abstract

**Background**: Carcinogenic polycyclic aromatic hydrocarbons (PAHs), particularly benzo[a]pyrene (BaP) and the EFSA-recommended PAH4 index (benzo[a]pyrene, benzo[a]anthracene, benzo[b]fluoranthene, and chrysene), can accumulate in human breast milk following maternal exposure. **Aim**: This study aimed to determine BaP and PAH4 levels in breast milk samples of lactating mothers in Hungary and to evaluate demographic, lifestyle, dietary, and environmental predictors of these carcinogenic PAHs. **Methods**: Breast milk samples (n = 50) were analyzed using high-performance liquid chromatography with fluorescence detection (HPLC/FLD). Due to the right-skewed distribution of PAH concentrations, BaP and PAH4 values were log10-transformed before statistical analysis. Associations between log10-transformed PAH concentrations and maternal variables were assessed using *t*-tests, Pearson correlation, and multivariable linear regression. **Results**: BaP was detected in 32 of 50 samples (64%), whereas PAH4 was detectable in 44 of 50 samples (88%). Frequent milk consumption was independently associated with higher log10-transformed PAH4 concentrations in multivariable analysis. Samples collected during the heating season tended to show higher carcinogenic PAH levels compared to those obtained outside the heating period. Other demographic and lifestyle factors showed no consistent independent associations. **Conclusions**: Lifestyle and environmental factors, particularly dietary habits and seasonal exposure, contribute to carcinogenic PAH levels in breast milk among Hungarian mothers. Identifying modifiable determinants may support strategies to reduce infant exposure.

## 1. Introduction

Polycyclic aromatic hydrocarbons (PAHs) are ubiquitous environmental contaminants formed during the incomplete combustion of organic materials, including fossil fuels, biomass, and tobacco [1,2,3,4]. Human exposure occurs primarily through inhalation of polluted air, dietary intake, and dermal contact [2]. Several PAHs exhibit carcinogenic and mutagenic properties, and high-molecular-weight PAHs (4–6 rings) are generally considered the most toxic [2]. Among these compounds, benzo[a]pyrene (BaP) is recognized as a potent carcinogen, and the EFSA CONTAM Panel recommends the PAH4 group—benzo[a]anthracene (BaA), chrysene (CHR), benzo[b]fluoranthene (BbF), and BaP—as an indicator of carcinogenic PAH contamination in food [1]. PAH4 is considered a more robust marker of carcinogenic PAH contamination than BaP alone, as it reflects carcinogenic PAHs relevant for exposure assessment [1].

Due to their lipophilic nature, PAHs can accumulate in adipose-rich tissues and may be transferred into human breast milk following maternal exposure [4]. Consequently, breastfed infants may be exposed to these compounds during a critical developmental period. Previous studies and systematic reviews have reported detectable concentrations of PAHs in breast milk, with variability linked to environmental pollution, indoor heating, cooking practices, maternal lifestyle, and dietary habits [4,5,6,7,8,9,10,11,12,13,14,15,16,17]. Early-life PAH exposure has been associated with adverse outcomes, including respiratory effects such as wheeze and asthma [5,18].

Hungary, similar to other Central European regions, experiences marked seasonal fluctuations in ambient PAH levels, driven mainly by domestic heating [2,3,11,12]. Such seasonal patterns are consistent with wintertime increases in combustion-related emissions (including biomass burning), which can contribute substantially to ambient carcinogenic PAH burdens [11,12]. Despite this, data on carcinogenic PAHs (BaP and PAH4) in human breast milk from Hungarian women remain limited, and determinants influencing these carcinogenic markers are not well characterized.

Dietary exposure pathways may also be relevant for carcinogenic PAHs, particularly through animal-derived, fat-containing foods. PAHs have been reported in milk and dairy products, and dairy processing and environmental contamination of feed or raw materials have been proposed as potential contributors to PAH occurrence in these products. In our previous study, total PAH concentrations in breast milk samples from Hungarian mothers were quantified, and carcinogenic risk to breastfed infants was estimated using the Incremental Lifetime Cancer Risk (ILCR) model [6]. However, that study did not specifically address determinants of carcinogenic PAH markers, such as BaP and PAH4. The present study builds on the same cohort and analytical dataset by focusing specifically on BaP and PAH4, the two indicators currently used in carcinogenic PAH risk assessment, and by evaluating their demographic, dietary, lifestyle, and environmental determinants. Identifying modifiable dietary and environmental determinants may be useful for developing targeted risk communication and exposure-reduction strategies for breastfeeding mothers [2,4]. Therefore, the present study aimed to investigate the occurrence and determinants of the carcinogenic PAH indicators BaP and PAH4 in breast milk samples obtained from Hungarian lactating mothers. To our knowledge, this is the first study to investigate the determinants of carcinogenic PAHs in breast milk among Hungarian women.

## 2. Materials and Methods

### 2.1. Study Population and Ethical Approval

The present study was based on the same cohort and analytical dataset described in our previous publication [6]. Briefly, lactating women from Hungary were recruited on a voluntary basis between 2014 and 2019. Participants were invited to provide a breast milk sample and complete a structured questionnaire covering demographic characteristics, dietary habits, lifestyle factors, and environmental exposures. Because of the practical challenges associated with collecting human breast milk samples, a convenience sampling approach was used. A total of 50 breast milk samples with complete analytical and questionnaire data were included in the present analyses.

This study included breast milk samples collected from 50 lactating mothers residing in different regions of Hungary. Participant recruitment was facilitated by the Hungarian Nursing Association (Magyar Védőnői Szolgálat), which assisted in contacting eligible volunteers. All participants were at least 18 years of age and provided written informed consent before sample collection.

Donors completed a structured questionnaire collecting information on demographic characteristics (age, education, place of residence), lifestyle factors (smoking status, dietary habits, consumption of organic foods), and anthropometric data, including pre-pregnancy body mass index (BMI). Detailed demographic characteristics of the study population have been reported previously [6]. Table 1 summarises variables relevant to the present analysis.

Breast milk collection was conducted in accordance with ethical approvals issued by the Ministry of Human Resources (decision number: 14069-6/2018/EÜIG) and the Regional Research Ethics Committee of the University of Pécs (approval number: 5579). All procedures were performed in compliance with the principles of the Declaration of Helsinki.

Further details regarding participant recruitment and sample collection have been reported previously [6].

The present study is based on the same cohort, breast milk sample set, and chemical analytical dataset as our previously published study [6]. No new sample collection or new chemical analysis was performed. The current manuscript represents a focused secondary analysis of the existing analytical dataset, restricted to the carcinogenic PAH markers BaP and PAH4, and evaluates their associations with demographic, dietary, lifestyle, and environmental variables.

### 2.2. Sample Collection and Storage

Approximately 10–20 mL of breast milk was collected either by manual expression or using sterile breast pumps, according to maternal preference. Samples were transferred into pre-cleaned amber glass vials, immediately cooled, and transported to the laboratory under refrigerated conditions. Upon arrival, samples were stored at −20 °C until analysis to minimise potential degradation of PAH compounds. Breast milk sampling was conducted between 2014 and 2019. PAH measurements were performed at the Institute of Bioanalysis, University of Pécs.

### 2.3. Chemicals and Reagents

The EPA 525 PAH standard mixture (500 μg/mL in dichloromethane), containing 13 polycyclic aromatic hydrocarbons (PAHs)—acenaphthylene (Ace), anthracene (Ant), benzo[a]anthracene (BaA), benzo[a]pyrene (BaP), benzo[b]fluoranthene (BbF), benzo[g,h,i]perylene (BghiP), benzo[k]fluoranthene (BkF), chrysene (CHR), dibenzo[a,h]anthracene (DBA), fluorene (FLU), indeno[1,2,3-c,d]pyrene (IP), phenanthrene (PHE), and pyrene (PYR)—was purchased from Sigma-Aldrich (St. Louis, MO, USA).

### 2.4. Sample Preparation and Chemical Analysis

Breast milk samples were thawed at 37 °C in the dark before extraction. PAHs were extracted from 5 mL of breast milk by liquid–liquid extraction using 3 mL of n-hexane. The mixture was vortexed, incubated at 37 °C, and centrifuged (3000 rpm, 10 min, 4 °C). The organic phase was collected and subjected to silica gel clean-up.

Silica columns were prepared by packing 2 g of silica gel and conditioning them with n-hexane: dichloromethane (3:1, *v*/*v*). The extracts were loaded onto the column, washed with n-hexane, and PAHs were eluted with n-hexane: dichloromethane (3:1, *v*/*v*). The eluates were evaporated to dryness under a gentle nitrogen stream and reconstituted in 1 mL of acetonitrile before analysis by high-performance liquid chromatography with fluorescence detection (HPLC-FLD).

Breast milk samples were analysed by high-performance liquid chromatography with fluorescence detection (HPLC-FLD) using a Shimadzu LC-20AD HPLC system (Shimadzu Corp., Kyoto, Japan) equipped with a binary solvent delivery system, an autosampler, a column heater, an RF-20A/RF-20Axs fluorescence detector (Hamamatsu Corp., Shizuoka, Japan), and a UV diode-array detector (DAD) (Hamamatsu Corp., Shizuoka, Japan).

All samples were extracted in duplicate. Method blanks prepared using distilled water were processed through the entire analytical procedure to evaluate potential background contamination. All organic solvents (hexane, cyclohexane, dichloromethane, and acetonitrile) were liquid chromatography–mass spectrometry (LC-MS) grade (VWR International, Radnor, PA, USA).

### 2.5. Method Validation

The analytical method was validated according to previously described procedures [6]. Calibration curves were constructed using nine concentration levels (0.0625–50 ng/mL) prepared in acetonitrile and analysed in triplicate. Method accuracy was evaluated using breast milk samples spiked at three concentration levels (5, 10, and 20 ng/mL). Quantification was performed using external standard calibration.

Limits of detection (LOD) and quantification (LOQ) were determined based on signal-to-noise ratios of 3:1 and 10:1, respectively. The method demonstrated good linearity for all target PAHs (R^2^ > 0.987). For the PAH4 compounds investigated in the present study, coefficients of determination ranged from 0.9978 to 0.9991, with mean recovery values between 79.5% and 95.9%. Overall, LOD and LOQ values ranged from 0.07 to 0.25 ng/mL and 0.21 to 0.76 ng/mL, respectively, while mean recovery values for all target PAHs ranged from 72.8% to 135.1%, depending on the analyte.

Representative chromatograms obtained using the same HPLC-FLD method and breast milk sample set have been published previously [6].

### 2.6. PAH4 Calculation and Handling of Non-Detects

PAH4 was calculated as the sum of benzo[a]pyrene (BaP), benzo[a]anthracene (BaA), chrysene (CHR), and benzo[b]fluoranthene (BbF). Concentrations below the limit of detection (LOD) were replaced by LOD/2 before PAH4 calculation and log10 transformation. LODs were 0.07 ng/mL for BaP, and for the other PAH4 components, 0.10 ng/mL (BaA), 0.08 ng/mL (CHR), and 0.09 ng/mL (BbF).

### 2.7. Statistical Analysis

Statistical analyses were performed using IBM SPSS Statistics version 29 (IBM Corporation, Armonk, NY, USA).

Values below the LOD were replaced by LOD/2, a widely used approach for handling left-censored environmental contaminant data [19], prior to PAH4 calculation and log10 transformation. Because BaP and PAH4 concentrations showed right-skewed distributions, log10 transformation was applied prior to statistical analysis. Descriptive statistics are presented as the mean ± standard deviation (SD) or the median with interquartile range (IQR), depending on the data distribution.

Normality of log-transformed PAH concentrations was assessed using the Shapiro–Wilk test and graphical inspection of Q–Q plots. Although slight deviations from normality were observed, parametric statistical tests were applied considering the sample size and approximate symmetry of the distributions.

Associations between log-transformed PAH concentrations and maternal demographic, lifestyle, dietary, and environmental variables were evaluated using independent samples *t*-tests, Pearson correlation analysis, and multivariable linear regression models.

Variables showing potential associations in univariate analyses were included in multivariable models to identify independent predictors of carcinogenic PAH concentrations. Statistical significance was defined as *p* < 0.05.

## 3. Results

### 3.1. Descriptive Statistics of BaP and PAH4

BaP was detected in 32 of 50 breast milk samples (64%). PAH4 was considered detectable when at least one of its four components (BaP, BaA, CHR, or BbF) was detected above the LOD; based on this definition, PAH4 was detectable in 44 of 50 samples (88%). Descriptive statistics are presented on the original concentration scale (Table 2), whereas statistical analyses were performed using log10-transformed data.

BaP concentrations showed a geometric mean (GM) of 0.09 ng/mL (median: 0.035 ng/mL; IQR: 0.035–0.23 ng/mL; range: 0.035–1.11 ng/mL). The corresponding PAH4 concentrations had a GM of 2.29 ng/mL (median: 3.175 ng/mL; IQR: 0.833–6.750 ng/mL; range: 0.17–18.05 ng/mL).

### 3.2. Maternal Demographic Determinants

Maternal age showed no significant association with log-transformed BaP (β = −0.080, *p* = 0.586) or PAH4 concentrations (β = −0.131, *p* = 0.370). Educational level (university degree vs. no degree) was not significantly associated with log-transformed BaP (t = 0.328, *p* = 0.744) or PAH4 concentrations (t = 0.222, *p* = 0.825). Pre-pregnancy BMI showed no significant association with log-transformed BaP (β = 0.109, *p* = 0.454) or PAH4 concentrations (β = −0.053, *p* = 0.719). No significant differences were observed between urban and rural participants regarding log-transformed BaP (t = −0.180, *p* = 0.858) or PAH4 concentrations (t = −0.444, *p* = 0.659).

### 3.3. Lifestyle and Dietary Factors

Fast food consumption was significantly associated with log-transformed PAH4 concentrations, with higher PAH4 levels observed in mothers reporting fast food consumption compared to non-consumers (t = −2.041, *p* = 0.047). No significant association was observed for BaP (*p* = 0.837).

Milk consumption frequency was also significantly associated with log-transformed PAH4 concentrations; mothers reporting frequent milk consumption showed higher PAH4 levels than those consuming milk rarely (t = −2.639, *p* = 0.012). Post-hoc comparisons across the four original milk-consumption categories indicated that this difference was primarily driven by daily milk consumption compared with rare/never consumption (*p* = 0.010). No statistically significant association was observed between milk consumption frequency and log-transformed BaP concentrations (*p* = 0.117).

No significant associations were observed between general meat consumption frequency, fatty food intake, or organic product consumption and log-transformed BaP or PAH4 concentrations (all *p* > 0.05).

### 3.4. Environmental Factors

Sampling season was significantly associated with log-transformed PAH4 concentrations. Samples collected during the heating season exhibited higher log10-transformed PAH4 concentrations compared to those collected outside the heating season (t = −2.052, *p* = 0.046) (Table 3). No significant seasonal difference was observed for log-transformed BaP concentrations (*p* = 0.778).

Only three mothers reported active smoking. Due to the very small number of smokers, the effect of smoking on PAH concentrations could not be reliably evaluated, although descriptively higher PAH4 levels were observed among smokers.

### 3.5. Multivariable Regression

A multivariable linear regression model was constructed to evaluate independent predictors of log10-transformed PAH4 concentrations, including milk consumption frequency, fast food consumption, heating season, and maternal age. The overall model was statistically significant (F = 3.299, *p* = 0.020) and explained 25.3% of the variance in log10-transformed PAH4 concentrations (adjusted R^2^ = 0.176).

**Figure 1:**
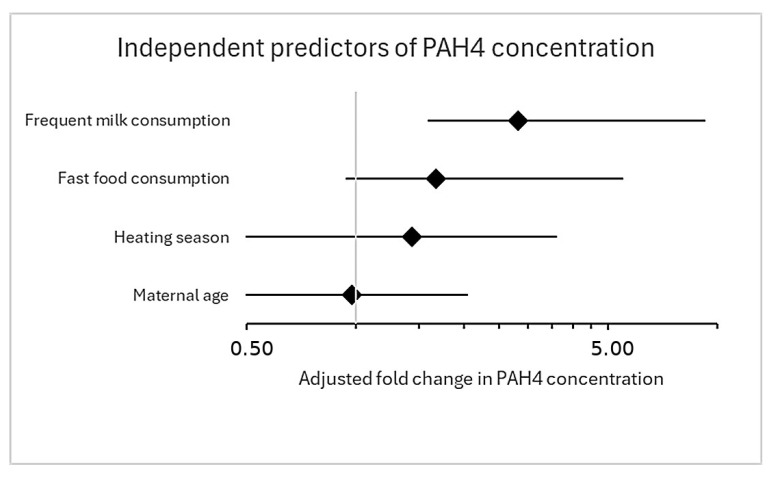
Forest plot showing adjusted fold changes (10^B) with 95% confidence intervals for independent predictors of PAH4 concentrations in multivariable linear regression analysis. The vertical line at 1 represents no association.

Frequent milk consumption remained independently associated with higher log10-transformed PAH4 levels (β = 0.355, *p* = 0.020). Heating season showed a positive but non-significant trend (β = 0.238, *p* = 0.099), while fast food consumption and maternal age were not independently associated with PAH4 concentrations (Table 4). Figure 1 presents a forest plot of adjusted fold changes (10^B) with 95% confidence intervals.

## 4. Discussion

### 4.1. Comparison with the Previous Study

This study builds on our previous nationwide assessment of PAH contamination in breast milk from Hungarian mothers by shifting the focus from the overall PAH burden (PAH12/total PAHs) to the carcinogenic indicators BaP and PAH4 [6]. BaP was originally adopted as a marker of carcinogenic PAH contamination, whereas PAH4 is currently recommended by EFSA as the preferred indicator of carcinogenic PAH exposure [1]. In the earlier work, total PAH concentrations showed wide variability [6]. They were dominated by lower-molecular-weight PAHs (e.g., fluorene, phenanthrene, pyrene) [6]. In contrast, BaP was detected at substantially lower concentrations—consistent with the general pattern that lower-molecular-weight PAHs are more prevalent in breast milk than higher-molecular-weight carcinogenic congeners [6].

From an exposure perspective, this distinction is relevant because determinants that influence overall PAH burden (which may often be driven by more ubiquitous, lower-molecular-weight PAHs) may not necessarily predict carcinogenic PAH markers, which may reflect more source-specific exposure contributions. In our previous analysis, total PAHs were higher among mothers living in urban settings [6]. They were associated with several dietary variables (including meat and dairy consumption) as well as lower consumption of organic products [6]. Smoking was rare (three self-reported smokers), limiting statistical inference, although higher mean levels were observed among smokers [6].

In contrast, no significant association between place of residence and BaP or PAH4 concentrations was observed in the present study. This finding suggests that the previously observed urban–rural difference in total PAH burden cannot be explained solely by differences in the carcinogenic PAH markers investigated here and may instead reflect differences in other PAH compounds contributing to the overall PAH burden.

In the present analysis, restricting outcomes to BaP and PAH4 and applying log10-transformed models resulted in fewer statistically significant associations. This is plausible given the lower concentration range and higher degree of left-censoring for BaP and because PAH4 captures only four carcinogenic PAHs rather than the full mixture. Notably, the most consistent finding in the current models was the association between higher milk consumption frequency and higher PAH4 levels. While this aligns with the biological plausibility highlighted previously—namely, the lipophilic nature of PAHs [1,13,14,15,16] and their potential to accumulate in animal-derived, fat-containing foods—other dietary determinants observed for total PAHs in the earlier study (e.g., meat consumption, organic food frequency) were not clearly associated with PAH4 in the present dataset [6].

A plausible explanation is that total PAHs were largely driven by more abundant, lighter compounds, whereas PAH4 reflects a narrower carcinogenic profile with potentially different source contributions. In addition, the use of log-scale modelling and LOD-based imputation may attenuate associations when non-detects are common, particularly for BaP. Environmental seasonality provides another link between the two studies. Although samples collected during the heating season showed higher PAH4 concentrations in the univariate analysis, this association was not retained in the multivariable model. This suggests that seasonality itself may not be an independent determinant of PAH4 concentrations but may instead reflect correlated behavioural, household, or environmental factors associated with wintertime exposure.

Overall, comparing the two studies suggests that determinants of overall PAH contamination and determinants of carcinogenic PAH markers partly overlap but are not identical. This distinction is relevant for exposure-focused risk communication: interventions aimed at reducing general PAH exposure may not always translate directly into lower levels of carcinogenic markers, and targeted guidance may therefore be needed.

### 4.2. Comparison with International Studies Focusing on BaP and PAH4

Beyond the comparison with our previous total PAH assessment, it is important to place the present findings within the international literature focusing on carcinogenic PAHs in breast milk. Studies from Europe and other regions have reported detectable levels of BaP and PAH4 components in human milk, although concentrations vary depending on environmental pollution, dietary habits, and combustion-related exposure sources [4,7,8,9,10].

The geometric mean BaP and PAH4 concentrations observed in our Hungarian cohort are broadly consistent with levels reported in European populations, where carcinogenic PAHs are typically present at lower concentrations than lighter PAHs but remain detectable in a substantial proportion of samples [4,7,8,9]. This suggests that lactating mothers in Hungary may experience background exposure to combustion-derived PAHs comparable to that reported in other European settings [2,11,12].

Importantly, international evidence indicates that determinants of carcinogenic PAHs may differ from those influencing total PAH burden. While lighter PAHs often reflect more diffuse environmental exposure, higher-molecular-weight carcinogenic PAHs are more closely linked to high-temperature combustion processes, including residential heating, traffic emissions, tobacco smoke, and certain cooking practices [2,11,12]. In this context, the seasonal trend and dietary association observed for PAH4 in the present study are in line with a source-specific exposure pattern for carcinogenic PAHs.

### 4.3. Interpretation of the Milk Consumption Signal

One of the key findings of the present study is the independent association between frequent milk consumption and higher PAH4 concentrations in breast milk. Given the lipophilic nature of PAHs, dietary intake via animal-derived, fat-containing foods—particularly dairy—represents a plausible exposure pathway. PAHs have been reported in milk and dairy products, and potential sources include environmental contamination of feed or raw materials, as well as processing-related contamination [13,14,15,16].

The absence of a similar association for meat consumption in the present carcinogenic-focused analysis, despite its relevance in our previous total PAH assessment, suggests that specific dietary items may contribute differently to distinct PAH profiles [6]. In addition, milk consumption frequency may capture broader dietary patterns or lifestyle factors that were not fully quantified in the questionnaire.

A further consideration is statistical: this study evaluated log-transformed carcinogenic markers in a modest sample size, and BaP showed a proportion of non-detects requiring LOD-based imputation. Under these conditions, weaker dietary associations—particularly for BaP—may be more difficult to detect in multivariable models.

Overall, while these findings should be interpreted cautiously, they support the relevance of dietary pathways—particularly those involving milk consumption—as potential contributors to carcinogenic PAH exposure during lactation [13,14,15,16].

### 4.4. Environmental Determinants and Seasonality

Seasonal variation represents another biologically plausible determinant of carcinogenic PAH levels. In Central European regions, including Hungary, residential heating during colder months substantially increases ambient concentrations of combustion-derived PAHs [2,3,11,12]. Although the heating season variable did not reach statistical significance in all analyses, its direction of association suggests that wintertime exposure may contribute to elevated PAH4 levels.

This observation is consistent with known seasonal patterns in atmospheric PAH emissions and supports the role of combustion-related environmental sources in shaping breast milk contamination profiles [11,12].

### 4.5. Strengths and Limitations

This study has several strengths. To our knowledge, it represents the first investigation specifically focusing on carcinogenic PAH markers (BaP and PAH4) in breast milk among Hungarian mothers. The use of a previously validated HPLC-FLD method, standardized sample preparation, and log-transformed multivariable modelling supports analytical reliability [6]. Moreover, examining determinants rather than solely reporting concentrations provides a more exposure-oriented perspective.

However, certain limitations must be acknowledged. The sample size (n = 50) limits statistical power, particularly when evaluating multiple dietary and lifestyle variables. The proportion of non-detects for BaP required LOD-based imputation, which may attenuate associations. Smoking prevalence was low (three self-reported smokers), preventing meaningful statistical evaluation of this well-established PAH source. Additionally, dietary information was based on self-reported frequency data rather than quantitative intake assessment, which may introduce misclassification.

Another limitation of the present study is that information on infant age and duration of lactation was not collected. As lactation duration may influence breast milk composition and the transfer of lipophilic contaminants, its potential effect on PAH concentrations could not be evaluated and should be considered in future studies.

Finally, multiple comparisons were performed in exploratory analyses; therefore, observed associations should be interpreted with caution and considered hypothesis-generating.

## 5. Conclusions

Taken together, the present findings refine our understanding of PAH contamination in human breast milk in Hungary. While total PAH burden reflects a broad mixture influenced by multiple environmental and lifestyle factors, carcinogenic markers such as PAH4 appear to be more selectively influenced by specific dietary habits—most notably frequent milk consumption—and seasonal exposure conditions. This distinction is relevant for exposure-focused public health communication, emphasizing that not all PAHs behave identically in terms of determinants and potential health implications.

## Figures and Tables

**Table 1:** Selected demographic characteristics of the participating mothers.

Demographic Data of Participating Women (Number of Data)		Frequency (Percentage)
Residence (n = 50)	Rural	18 (36%)
	Urban	32 (64%)
Education level (n = 47)	Primary education	5 (10.6%)
	Vocational education	8 (17%)
	Secondary education	14 (28%)
	Higher education	20 (42.6%)
Age of mothers (n = 50)	Mean age ± SD (years): 29.3 ± 5.2	
Pre-pregnancy BMI (n = 48)	Underweight	3 (6.3%)
	Normal	28 (58.3%)
	Overweight	10 (20.8%)
	Obese (BMI ≥ 30 kg/m^2^)	7 (14.7%)
Postpartum BMI (n = 48)	Underweight	2 (4.2%)
	Normal	21 (43.8%)
	Overweight	11 (22.9%)
	Obese (BMI ≥ 30 kg/m^2^)	14 (29.3%)
Parity (n = 48)	Primiparous	24 (50%)
	Multiparous	24 (50%)

**Table 2:** Descriptive statistics of BaP and PAH4 concentrations in breast milk samples (ng/mL).

Compound	Detection Frequency n/N (%)	Mean ± SD	Median (IQR)	Range	Geometric Mean (GM)
BaP	32/50 (64%)	0.172 ± 0.235	0.035 (0.035–0.23)	0.035–1.11	0.09
PAH4	44/50 (88%)	4.468 ± 4.196	3.175 (0.833–6.750)	0.17–18.05	2.29

Detection frequency refers to concentrations above the LOD. LODs were 0.07 ng/mL for BaP, 0.10 ng/mL for BaA, 0.08 ng/mL for CHR, and 0.09 ng/mL for BbF. Statistical analyses were performed using log10-transformed concentration data.

**Table 3:** Univariate associations between selected dietary and environmental factors and PAH4 concentrations in breast milk.

Variable	Category	N	PAH4 (GM, ng/mL)	*p*-Value
Fast food consumption	No	26	1.53	0.047
	Yes	19	3.52	
Milk consumption frequency	Rare (never/occasionally)	21	1.23	0.012
	Frequent (weekly/daily)	24	3.60	
Heating season	Non-heating season	33	1.73	0.046
	Heating season	16	4.12	

Values are presented as geometric mean (GM, ng/mL). Statistical comparisons were performed using independent-samples *t*-tests on log10-transformed PAH4 concentrations.

**Table 4:** Multivariable linear regression analysis of factors associated with log10-transformed PAH4 concentrations in breast milk.

Predictor	B (SE)	Standardized β	*p*-Value
Heating season (yes vs. no)	0.157 (0.093)	0.238	0.099
Fast food consumption (yes vs. no)	0.223 (0.184)	0.175	0.234
Maternal age (years)	−0.009 (0.019)	−0.067	0.646
Frequent milk consumption (frequently vs. rarely)	0.448 (0.185)	0.355	0.020

Dependent variable: log10-transformed PAH4 concentration. Variables were entered simultaneously (enter method).

## Data Availability

The data supporting the findings of this study are available from the corresponding author upon reasonable request. The data are not publicly available due to ethical and privacy restrictions related to human-derived samples and participant information.

## Abbreviations

BaA	benzo[a]anthracene
BaP	benzo[a]pyrene
BbF	benzo[b]fluoranthene
BMI	body mass index
CHR	chrysene
CI	confidence interval
EFSA	European Food Safety Authority
FLD	fluorescence detector
GM	geometric mean
HPLC-FLD	high-performance liquid chromatography with fluorescence detection
IQR	interquartile range
LC-MS	liquid chromatography–mass spectrometry
LOD	limit of detection
LOQ	limit of quantification
PAH	polycyclic aromatic hydrocarbon
PAH4	benzo[a]anthracene + chrysene + benzo[b]fluoranthene + benzo[a]pyrene
SD	standard deviation
